# Cor triatriatum with dual fenestrations: delineation by cardiovascular magnetic resonance flow imaging

**DOI:** 10.1093/ehjimp/qyaf133

**Published:** 2025-10-24

**Authors:** Ahsan A Khan, Louis Kolman, James A White

**Affiliations:** Stephenson Cardiac Imaging Centre, University of Calgary, Foothills Medical Centre, #0700, SSB, 1403-29th St. NW, Calgary, Alberta, T2N2T9, Canada; Libin Cardiovascular Institute, University of Calgary, 2500 University Drive NW, Calgary, Alberta, T2N 1N4, Canada; Stephenson Cardiac Imaging Centre, University of Calgary, Foothills Medical Centre, #0700, SSB, 1403-29th St. NW, Calgary, Alberta, T2N2T9, Canada; Libin Cardiovascular Institute, University of Calgary, 2500 University Drive NW, Calgary, Alberta, T2N 1N4, Canada; Stephenson Cardiac Imaging Centre, University of Calgary, Foothills Medical Centre, #0700, SSB, 1403-29th St. NW, Calgary, Alberta, T2N2T9, Canada; Libin Cardiovascular Institute, University of Calgary, 2500 University Drive NW, Calgary, Alberta, T2N 1N4, Canada; Department of Diagnostic Imaging, Cumming School of Medicine, University of Calgary, Calgary, Alberta, Canada

## Case description

A 53-year-old man presented with chest pain and was diagnosed with non-ST elevation myocardial infarction (December 2024). He underwent percutaneous coronary intervention to the left anterior descending artery. At presentation, he also reported exertional dyspnoea and reduced exercise tolerance, although no symptoms specifically suggestive of pulmonary venous obstruction were noted.

Transthoracic echocardiography showed mildly reduced left ventricular systolic function (EF 50%) with apical akinesis. An incidental linear echo density in the left atrium suggested a remnant fibromuscular membrane. Colour Doppler demonstrated flow acceleration through the membrane with a mean gradient of 2 mmHg. Fenestrations were not clearly visualized on echocardiography, likely due to the thin, mobile nature of the membrane and limited acoustic windows.

Cardiac magnetic resonance (CMR) was pursued to clarify the anatomy and exclude associated congenital anomalies. Cine steady-state free precession (SSFP) imaging in multiple planes demonstrated a membrane partially dividing the left atrium, with a visible fenestration, suggesting possible cor triatriatum sinister (see Supplementary data online, *Video S1*). Further assessment by saturation band gradient echo cine (see Supplementary data online, *Video S2*) and phase contrast imaging (see Supplementary data online, *Videos S3* and *S4*) revealed two discrete flow jets across the membrane that were not detected by echocardiography due to superior tissue contrast and multiplanar capability of CMR. Mild mitral regurgitation was also noted, consistent with echocardiographic findings. No other congenital anomalies, such as atrial septal defect or persistent superior vena cava, were identified. The patient had no history of atrial fibrillation.

**Figure 1:**
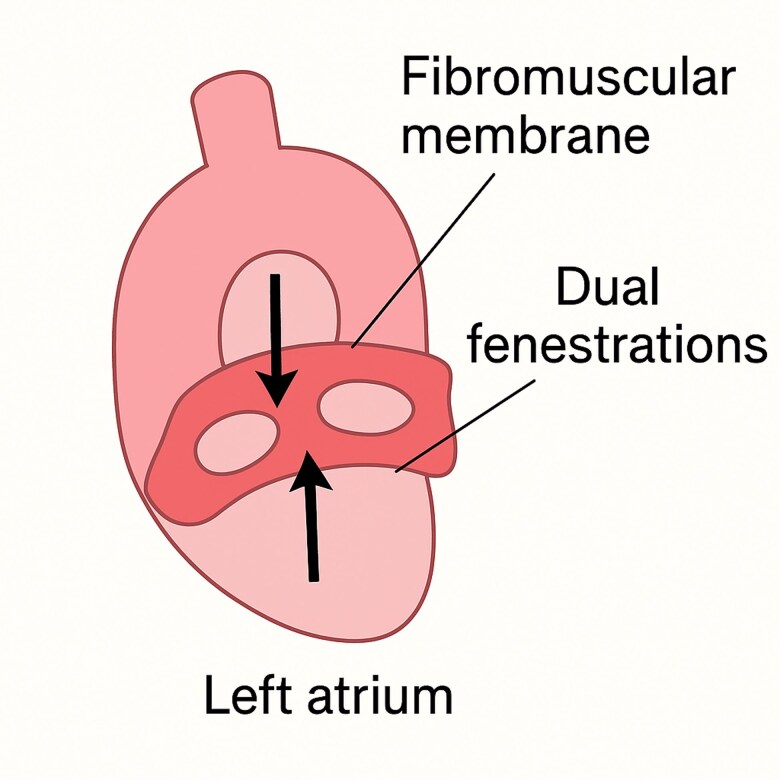
Schematic diagram of the left atrium showing a fibromuscular membrane with dual fenestrations (arrows indicate blood flow across the fenestrations).

Cor triatriatum is a rare congenital anomaly (*Figure 1*), accounting for <0.1% of congenital heart disease, and results from incomplete incorporation of the common pulmonary vein into the left atrium during embryogenesis. Clinical presentation depends on the size and number of fenestrations, ranging from asymptomatic to pulmonary venous obstruction. Diagnosis is often made by echocardiography but may be limited when fenestrations are small or multiple.

Previous reports have highlighted the incremental role of CMR in the evaluation of cor triatriatum, particularly when echocardiographic windows are limited or when fenestrations are small or multiple.^1,2^ Multimodality imaging reduces the risk of diagnostic pitfalls and is crucial for distinguishing obstructive from non-obstructive physiology, a distinction with direct implications for management strategies. In our case, CMR provided comprehensive structural and functional assessment beyond echocardiography, confirming non-obstructive dual fenestrations and guiding conservative management.

## Supplementary Material

qyaf133_Supplementary_Data

## Supplementary data

**Data availability:** No new data were generated or analysed in support of this research.

## Lead author biography

Dr Ahsan Aftab Khan, MBChB MRCP(UK) MD, is a cardiologist with subspecialty training in echocardiography and CMR imaging. He completed a two-year cardiac imaging fellowship in the UK covering echocardiography, cardiac CT, and MRI, followed by a Level 3 CMR fellowship in Calgary, Canada. He holds BSE and EACVI accreditations in echocardiography and CMR, and has presented imaging research at major international conferences. His clinical and academic interests include multimodality imaging in cardiomyopathy and valvular heart disease, with a strong focus on integrating advanced imaging into clinical decision-making.

## References

[1] Bockeria OL, Makhachev OA, Khiriev TK, Abramyan MA. Cor triatriatum in adults: diagnostic role of cardiovascular magnetic resonance imaging. Eur Heart J Cardiovasc Imaging 2013;14:151–7.

[2] Alphonso N, Nørgaard MA, Newcomb A, d'Udekem Y, Brizard CP, Cochrane A. Cor triatriatum: presentation, diagnosis and long-term surgical results. Ann Thorac Surg 2005;80:1666–71. 16242436 10.1016/j.athoracsur.2005.04.055

